# High-Resolution Ultrasound in Breast Cancer Diagnosis: Sensitivity, Specificity, and Clinical Implications

**DOI:** 10.7759/cureus.88641

**Published:** 2025-07-24

**Authors:** Aeimen Khalid, Muhammad Ahmad Mukhtar, Amna Mukhtar, Rubina Mukhtar

**Affiliations:** 1 General Medicine, Peterborough City Hospital, Peterborough, GBR; 2 General Medicine, York District Hospital, York, GBR; 3 Radiology, Nishtar Medical University, Multan, PAK; 4 Internal Medicine, University of Debrecen, Debrecen, HUN; 5 Radiology, MINAR (Multan Institute of Nuclear Medicine and Radiotherapy) Cancer Hospital, Multan, PAK

**Keywords:** breast, cancer, high-resolution ultrasound, negative predictive value, positive predictive value, sensitivity, specificity

## Abstract

Objective

This study aimed to explore the application of high-resolution ultrasound (HR US) in breast evaluation, with a focus on its sensitivity and specificity for the accurate characterization of breast lesions and the early detection of breast cancer.

Materials and methods

All patients presenting to the breast imaging department of MINAR Cancer Hospital, Multan, Pakistan, with clinically palpable breast lesions were included in this prospective cross-sectional study conducted from January 2023 to December 2023.

Results

In total, 6150 patients were registered. Of these, 242 were found to have different breast lesions on mammography and HR US. Lesions were categorized on the Breast Imaging Reporting and Data System (BI-RADS) scale. All were subjected to biopsies for histopathology to confirm the final diagnosis. Results were compared with those of histopathology. The true negative rate for malignancy in BI-RADS 2 and 3 lesions is 98.8% and 80%, respectively. The true positive rate for malignancy in BI-RADS 5 lesions is 99.14%, whereas for BI-RADS 4 lesions, it is only 12.5%. Specificity for BI-RADS 4 and 5 is 83.33% and 99.13%, respectively. Sensitivity for BI-RADS 2 and 3 lesions is 98.7% and 30%.

Conclusion

HR US has significant value in the characterization of breast lesions and holds particular importance in differentiating benign from malignant lesions, especially in situations where mammography is either unavailable or contraindicated. Additionally, it aids in the selection of patients for invasive procedures such as biopsies.

## Introduction

As the most common cancer among women and a leading cause of mortality, particularly in developing countries [1,2], breast cancer continues to be a subject of significant concern and ongoing discussion. Awareness campaign for the early detection of breast lesions by screening and self-breast examination is a primary focus worldwide in an effort to control its mortality rate [3]. The effectiveness of this campaign to raise awareness is unquestionable, but it also created a phobia of developing breast cancer, especially in young women. This phobia is a leading cause of an increasing number of patients presenting to breast care clinics and breast imaging departments for mammography to rule out cancer, causing a burden on already compromised diagnostic setups of developing countries and financial constraints on patients for unnecessary diagnostic and invasive procedures of biopsies. Moreover, in our already strained and compromised healthcare system, mammography services and specialized expertise are not universally available [3,4]. The facility of dedicated breast care clinics equipped with breast imaging modalities with interventional radiology and qualified and experienced manpower is available only in a few renowned healthcare centers [5]. A parallel campaign emphasizing that not every breast lesion signifies cancer is a crucial necessity. There is a spectrum of different lesions affecting the breast [6]. However, diagnostic evaluation of any clinical presentation is imperative to rule out malignancy [3,7].

We conducted a prospective study to characterize and differentiate benign and malignant lesions on high-resolution ultrasound (HR US) to determine both its sensitivity and specificity and the morphology and prevalence of different breast lesions in our population.

## Materials and methods

Study design and duration

This descriptive cross-sectional study was conducted from January 2023 to December 2023. All patients presented during this study period with some breast symptoms were enrolled and evaluated on a triple regimen policy based on clinical evaluation on history and examination, imaging, and tissue diagnosis by Trucut biopsies.

Study location

The study was conducted at the breast care clinic of MINAR (Multan Institute of Nuclear Medicine and Radiotherapy) Cancer Hospital, Multan, Pakistan. As the only public sector tertiary care cancer hospital in southern Punjab, this institution serves as a critical hub for oncology services. Its catchment area spans the entirety of southern Punjab and extends into upper Punjab, including Faisalabad, as well as regions of Khyber Pakhtunkhwa, such as Dera Ismail Khan, and parts of Balochistan up to Loralai. Collectively, it provides essential cancer care to an estimated population of over 60 million people. It is a running full-time breast care clinic with 100% dedicated female staff and all basic facilities for the screening and diagnosis of breast cancer, including digital mammography, HR US, and invasive procedures of US-guided fine needle aspiration cytology (FNAC), Trucut biopsy, intralesional clipping, and intralesional wire localization of impalpable masses. Women from the whole catchment area with different breast complaints present here, giving geographical diversity and making the study comprehensive.

Inclusion criteria

The study included patients of all age groups with underlying breast pathology categorized on the Breast Imaging Reporting and Data System (BI-RADS) scale from 2 to 5. All were evaluated on HR US for the characterization of lesions, followed by Trucut biopsies to validate the ultrasonographic diagnosis of lesions. HR US was preceded by mammography in patients over 35 years of age, while HR US was the elemental choice for younger patients under 35 years of age.

Exclusion criteria

Excluded were patients on follow-up at that time who were diagnosed with different breast lesions, patients with BI-RADS 6 lesions, those who refused Trucut biopsies, and all patients with incomplete or missing data, including histopathology reports or imaging reports.

Ethical considerations

Approval from the relevant authorities and Ethical Committee of MINAR (Multan Institute of Nuclear Medicine and Radiotherapy) Cancer Hospital (approval number: M-3(13)/2018) was obtained prior to the commencement of data collection. The study was carried out with the patients' consent, obtained both verbally and in writing. Participants were given comprehensive information regarding the study's purpose and objectives. Additionally, the confidentiality of their personal information was maintained in accordance with IRB guidelines.

Study size

We adopted a consecutive sampling approach, and the sample size was not calculated a priori. Instead, all eligible patients who presented during the predefined study period were consecutively enrolled.

Potential source of bias

The total number of patients in both groups was equal, so there is no potential source of bias.

## Results

In total, 6150 patients presented to the breast clinic and imaging department during the study period. Common presenting symptoms included a lump, nipple discharge, and pain. The mean age of patients was 34.9 STD±11.46 years. Two thousand seven hundred patients were subjected to mammography. HR US was conducted on 3450 patients. Out of these, 242 patients were found to have some lesions that were characterized based on various features, including (1) shape (round, oval, or irregular), (2) orientation (wider than taller or taller than wider), (3) margins (regular, lobulated, irregular, well-circumscribed, or ill-defined), (4) echogenicity (hypoechoic, isoechoic, hyperechoic, or anechoic), (5) presence of posterior acoustic enhancement or shadowing, (6) calcifications, and (7) status of axillary lymph nodes.

After characterization, lesions were graded on the BI-RADS scale defined by the American College of Radiology (ACR) as follows: 0 for inconclusive studies, 1 for normal findings, 2 for benign lesions, 3 for probably benign lesions, 4 for probably suspicious lesions, and 5 for highly suspicious lesions. If the patient was already a biopsy-proven case of malignancy, the lesion was graded as BI-RADS 6.

**Figure 1:**
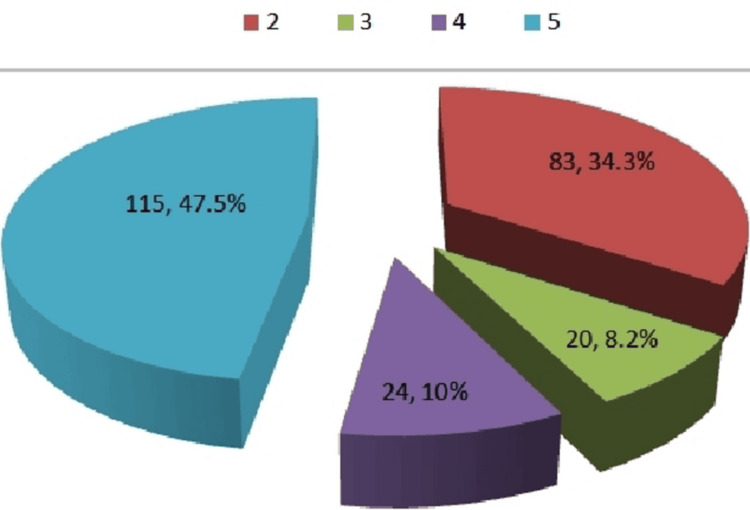
Frequency of different lesions on HR US categorized on BI-RADS HR US: high-resolution ultrasound; BI-RADS: Breast Imaging Reporting and Data System

The prevalence and frequencies are expressed in percentages. The prevalence of lesions categorized by BI-RADS is displayed in Figure 1. Imaging features of inflammatory lesions usually mimic those of cancers and show ill-defined irregular margins, so they are put in BI-RADS 4.

Trucut biopsies for tissue diagnosis were conducted on 242 patients, while others refused the procedure. Frequency of different breast lesions on tissue diagnosis is shown in Table 1.

**Table 1:** Comparison of diagnosis on HR US with histopathology HR US: high-resolution ultrasound; BI-RADS: Breast Imaging Reporting and Data System

BI-RADS on US	Type of lesions on tissue diagnosis	Number of lesions	Percentage
2	Fibroadenomas	68	82
	Benign breast parenchyma (focal thickening)	10	12
	Fibrocystic changes	4	4.8
	Atypical/suspicious cells	1	1.2
	Total	83	100
3	Fibroadenoma	16	80
	Malignancy	4	20
	Total	20	100
4	Inflammatory	20	83.3
	Malignancy	3	12.5
	Benign	1	4.2
	Total	24	100
5	Malignancy	114	99.13
	Benign	1	0.87
	Total	115	100

The results were assessed by comparing the diagnoses obtained from HR US and histopathology with tissue diagnosis from histopathology as gold standard. Each lesion was classified as either a true positive or a false positive based on histopathology results, which were categorized as benign or malignant. A true positive was defined as a lesion where the biopsy confirmed malignancy, while a false positive referred to a lesion where the biopsy revealed benign results.

After determining the numbers of true positives (TP), false positives (FP), true negatives (TN), and false negatives (FN), sensitivity, specificity, and positive predictive value (PPV) were calculated for BI-RADS 4 and 5, while negative predictive value (NPV) was calculated for BI-RADS 2 and 3 using the following respective standard formulas: for sensitivity, TP/TP+FN; for specificity, TN/TN+FP; for PPV, TP/TP+FP; and for NPV, TN/TN+FN.

**Figure 2:**
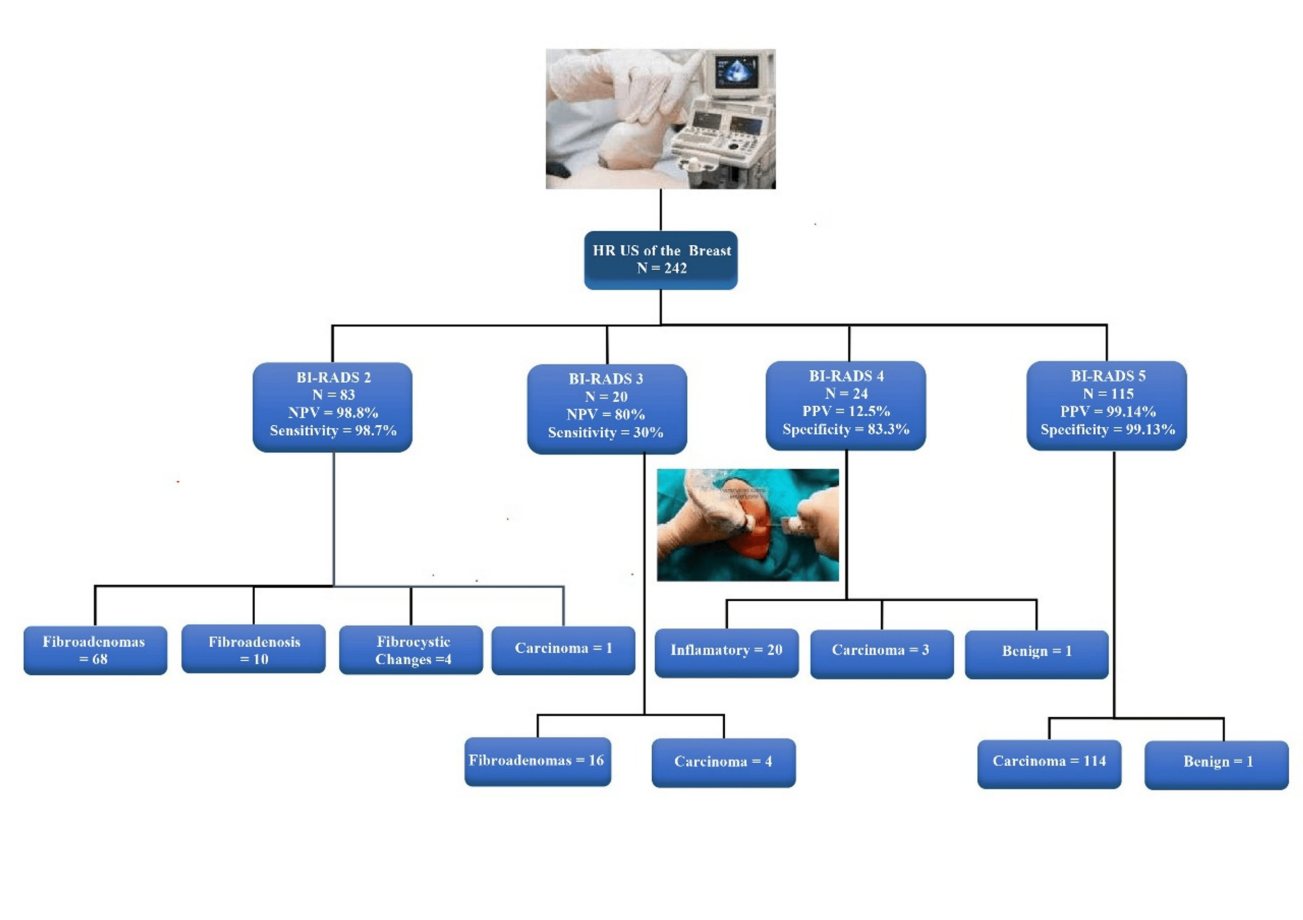
Flowchart summarizing the results HR US: high-resolution ultrasound; BI-RADS: Breast Imaging Reporting and Data System; NPV: negative predictive value; PPV: positive predictive value

NPV for malignancy in BI-RADS 2 and 3 lesions was 98.8% and 80%. PPV for malignancy in BI-RADS 5 lesions was 99.14%, while for BI-RADS 4 lesions, it was only 12.5%. Specificity for BI-RADS 4 and 5 is 83.33% and 99.13%, respectively. Sensitivity for BI-RADS 2 and 3 lesions is 98.7% and 30%. A chi-squared test of independence was conducted to validate the diagnostic accuracy of US results in comparison to tissue diagnoses. The analysis revealed no significant relationship between these variables. The chi-squared statistic is 0.4845, with a p-value of 0.922279. This result is not considered significant at the p<0.05 level. Equivalence testing using a predefined margin of ±10% indicated that both sensitivity and specificity of HR US fall well within clinically acceptable bounds when compared to tissue diagnosis. These findings support the potential of HR US as a reliable diagnostic tool in breast lesion assessment, particularly in settings where access to histopathology may be limited or delayed. The summary of results is shown in a quick view flowchart in Figure 2.

## Discussion

Evaluation of breast lesions by a single modality is neither revealing nor recommended. The internationally established protocol for the diagnosis of breast lesions is a triple regimen assessment based on a combination of clinical examination imaging and tissue diagnosis [8,9]. Mammography, HR US, nuclear scintimammography, and magnetic resonance imaging (MRI) are different imaging modalities used to divulge the breast lesion [6]. The role of mammography is imperative for both screening and diagnostic purposes [5,10]. In women over 35 years of age, the modality of choice is mammography. One of the major drawbacks of mammography is that its diagnostic accuracy depends on tissue density. It has a low sensitivity for the detection of small lesions (<2 cm) in dense breast, especially in young patients [5,11], and carries a 10% incidence of false negative report that further increases in case of dense breasts, thus limiting its use. Moreover, mammography often has difficulty distinguishing between solid and cystic lesions and sometimes between malignant and inflammatory lesions. To overcome these challenges, mammography is used in combination with other modalities, for example, HR US or MRI, in high-risk patients to enhance diagnostic efficiency [9]. Multiple studies utilizing both US and mammography have shown that the integration of these modalities yields an almost 100% predictive value for palpable breast lesions. This high level of accuracy underscores the importance of utilizing both imaging techniques in clinical practice to enhance diagnostic confidence [9].

US, being noninvasive, cost-effective [12], radiation-free, and independent of breast density, is a modality of choice in young and high-risk patients with dense breasts [4,5]. Over and above, in developing countries, a mammography facility is not freely available in remote areas or primary and secondary healthcare centers, especially in our developing country, but an HR US facility is available in the majority of primary and secondary care hospitals. 

Initially, breast US was the primary modality used to differentiate between solid and cystic lesions detected on mammography. However, advancements in US technology have made it the preferred tool for the initial evaluation of breast lesions, particularly in young, pregnant, and lactating women [10,13]. While it may not be possible to differentiate all benign solid breast nodules from malignant ones using US criteria, a practical objective for breast US is to identify a subset of solid nodules with a low risk of malignancy, including BI-RADS 3, so that short-interval follow-up can be considered a reasonable alternate to biopsy [14]. Studies demonstrated that these cases can be effectively managed with short-term follow-up every six months for two years [15-17]. Our study supports the use of HR US in distinguishing benign nodules from malignant ones, helping to determine whether further management through biopsy or follow-up is necessary.

Variable results on the efficacy of US accuracy for the characterization of breast lesions are shown in different studies. Consistent evidence from five different studies reveals that breast cancer screening using US exhibits high sensitivity (68.9-100%) and accuracy (0.687-0.999) compared to mammography alone. However, breast US has a lower specificity than mammography, with rates ranging from 22% to 99.9%. The PPV varies from 4.3% to 70%, while the NPV ranges from 61.2% to 100%. Heinig et al. also reported that the characterization of breast lesions using BI-RADS US criteria via US was highly accurate [15,18,19]. However, in a study based on the characterization of breast masses according to BI-RADS US criteria, Kwak et al. found no statistical differences between FNAC and US with regard to sensitivity and NPV (p>0.05) [20]. Our study demonstrates an NPV of 98.8% for BI-RADS 2 lesions and 80% for BI-RADS 3 lesions, which closely aligns with the figures reported in the literature, but the sensitivity and specificity decrease with BI-RADS 4 lesions, which may be attributed to the overlapping imaging features of inflammatory and malignant lesions. In such cases, a tissue biopsy is highly recommended and gains significance for accurate diagnosis.

In Chen et al.'s study comparing imaging modalities for detecting tumors smaller than 2 cm in breast cancer, it was found that US has a sensitivity of 85.1% for detecting tumors under 1 cm and 92.1% for tumors between 1.1 and 2 cm. US is particularly advantageous for identifying small breast cancers and is not affected by breast density [21], which corresponds to our findings.

Our study reveals high sensitivity and specificity for BI-RADS 2 and 5 lesions, respectively, while indicating low sensitivity and specificity for BI-RADS 3 and 4 lesions. This underscores the importance of considering tissue diagnosis for all BI-RADS 3 and 4 lesions. We wanted to study whether HR US and tissue diagnosis differ significantly in reliability and got an insignificant p-value. It means the two are nearly as reliable. The diagnostic accuracy falls within a 10% equivalence margin, indicating that HR US may be considered clinically equivalent to tissue diagnosis in this setting.

To summarize, breast US can be a valuable screening option for breast cancer, especially for young women with dense breast tissue. It is also beneficial in healthcare settings where mammography is not available [22]. Furthermore, it aids in characterizing clinically palpable breast lesions.

Limitations of the study

Breast cancer screening using US has advantages and limitations. The advantages of US include the following: (1) it is readily available, low cost, and affordable by the community; (2) it is independent of breast density; (3) results can be seen in real time; and (4) it is comfortable and safe to use because it does not use ionizing radiation, so repeated examinations can be done according to indications. Operator dependency and high recall rate are its limitations.

## Conclusions

Recent advancements in US technology enable a comprehensive approach to the diagnosis, management, and treatment of breast lesions. Effective utilization of this technology requires meticulous scanning techniques and careful attention to lesion morphology; however, the value of breast US is likely to continue growing and evolving.
